# Cystadenoma of Minor Salivary Glands With Cervical Metastasis: A Diagnostic Pitfall Revealing Underlying Cystadenocarcinoma

**DOI:** 10.7759/cureus.104309

**Published:** 2026-02-26

**Authors:** Mamadou Alpha Prateaux, Mohamed Amine Haouane, Issam Rharrassi, Mohamed Amine Azami

**Affiliations:** 1 Pathology, Cadi Ayyad University, Faculty of Medicine and Pharmacy, Avicenna Military Hospital, Marrakech, MAR; 2 Pathology, Cadi Ayyad University, Ibn Sina Military Hospital, Marrakech, MAR

**Keywords:** cervical lymph node metastasis, cystadenocarcinoma, cystadenoma, papillary, salivary

## Abstract

Papillary cystadenocarcinoma of the salivary glands is an exceptionally rare neoplasm. Despite advances in immunohistochemistry and molecular studies, the distinction between malignant and benign cystadenomatous tumors of salivary glands remains poorly defined. This case report details the pathological characteristics, highlighting the diagnostic difficulties and underscoring the importance of precise differentiation to guide effective clinical interventions.

We report the case of a 58-year-old woman who was initially diagnosed with papillary cystadenoma of a minor salivary gland. Two years later, she developed a cervical lymph node (LN) mass. Clinical, radiological, and histopathological examinations confirmed metastasis to the cervical LNs, characterized by low-grade papillary cystadenocarcinoma originating from the salivary gland.

Despite its potential to recur and metastasize, all specimens should be thoroughly examined and included, even in low-grade cases. Patients diagnosed with salivary gland cystadenomas should be closely monitored for early signs of relapse and/or metastasis. Therefore, understanding these pathological nuances is essential for clinicians and pathologists. Analyzing the molecular differences between the localized and metastatic forms of this neoplasm would enhance our understanding and documentation of this exceptional case.

## Introduction

Salivary gland cancers account for approximately 15% to 30% of all salivary gland tumors [1]. Papillary cystadenomatous neoplasms are particularly rare. Differentiating between benign (cystadenoma) and malignant (cystadenocarcinoma) forms poses a significant diagnostic challenge due to overlapping clinical, radiological, histological, and immunohistochemical features. Histological differentiation relies on the identification of capsule infiltration and/or pronounced cytonuclear atypia, as well as vascular and perineural invasion in cystadenocarcinoma [2]. According to the fourth edition of the World Health Organization (WHO) Classification of Head and Neck 2017, it is classified as a subtype of nonspecific adenocarcinomas [3].

We present the rare case of a 58-year-old woman who was initially diagnosed with papillary cystadenoma of the minor salivary gland. Two years later, she developed a cervical lymph node (LN) mass. Clinical, radiological, and histopathological examinations confirmed metastasis to the cervical LN, which was characterized by low-grade papillary cystadenocarcinoma originating from the salivary gland. This case report details the pathological characteristics, highlights the diagnostic difficulties, and underscores the importance of precise differentiation to guide effective clinical interventions. Consequently, understanding these pathological nuances is essential for both clinicians and pathologists.

## Case presentation

A 58-year-old woman presented with a well-defined nodule on her right cheek, present for one year and gradually increasing in size. The patient had no history of fever, cough, or chest pain. She had no personal or family history of cancer. Otherwise, she was in good health, and a review of systems was unremarkable. Physical examination revealed a nodule measuring 1.8 × 1.5 cm, with a smooth surface. The surface was smooth. The underlying mucosa was normal, exhibiting no color change or ulceration. No palpable cervical lymph nodes were detected.

An excisional biopsy was then performed. The specimen was circumscribed and globular, measuring 1.8 × 1.5 × 0.8 cm. It was serially sectioned transversely at intervals of 3 mm to 4 mm. The cut surface was solid, with a cystic area. The cystic areas were filled with mucoid material. Deeper tissue layers were evaluated to thoroughly exclude the possibility of malignancy. The entire lesion was submitted for histopathological examination and processed into four tissue blocks.

**Figure 1:**
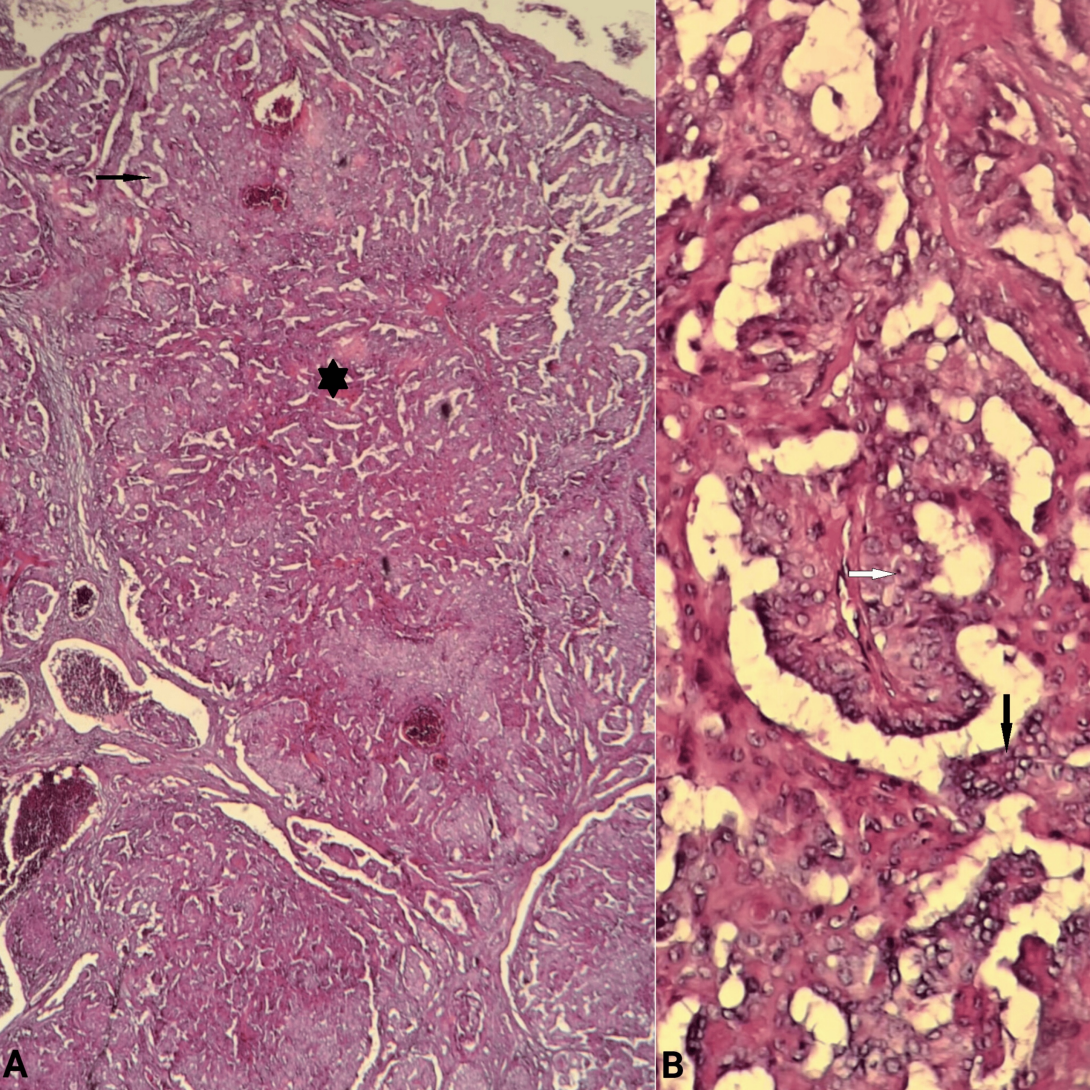
Papillary cystadenoma A: A well-defined, encapsulated, and cystic nodule lined by complex papillary fronds (black star) with fibrovascular cores (black arrow) (H&E stain, magnification x10); B: A double-cell layer of neoplastic epithelial cells exhibiting large, round, vesicular, and uniform nuclei (black arrow) with inconspicuous nucleoli (white arrow) (H&E stain, magnification x40)

Histopathological examination revealed a well-defined, encapsulated cystic nodule containing numerous cystic spaces lined by complex papillary fronds with fibrovascular cores. The inner layer consisted of cuboidal to columnar cells with eosinophilic and slightly granular cytoplasm. Their nuclei were large, round, vesicular, and uniform with inconspicuous nucleoli. Only a few cells displayed cytoplasmic vacuolation. The basal layer comprised cuboidal and flattened cells with darkly stained nuclei. No significant cytological atypia was observed. Mitotic activity was low. There was no evidence of necrosis, perineural infiltration, vascular or lymphatic invasion, or infiltration of the surrounding tissue (Figure 1).

**Figure 2:**
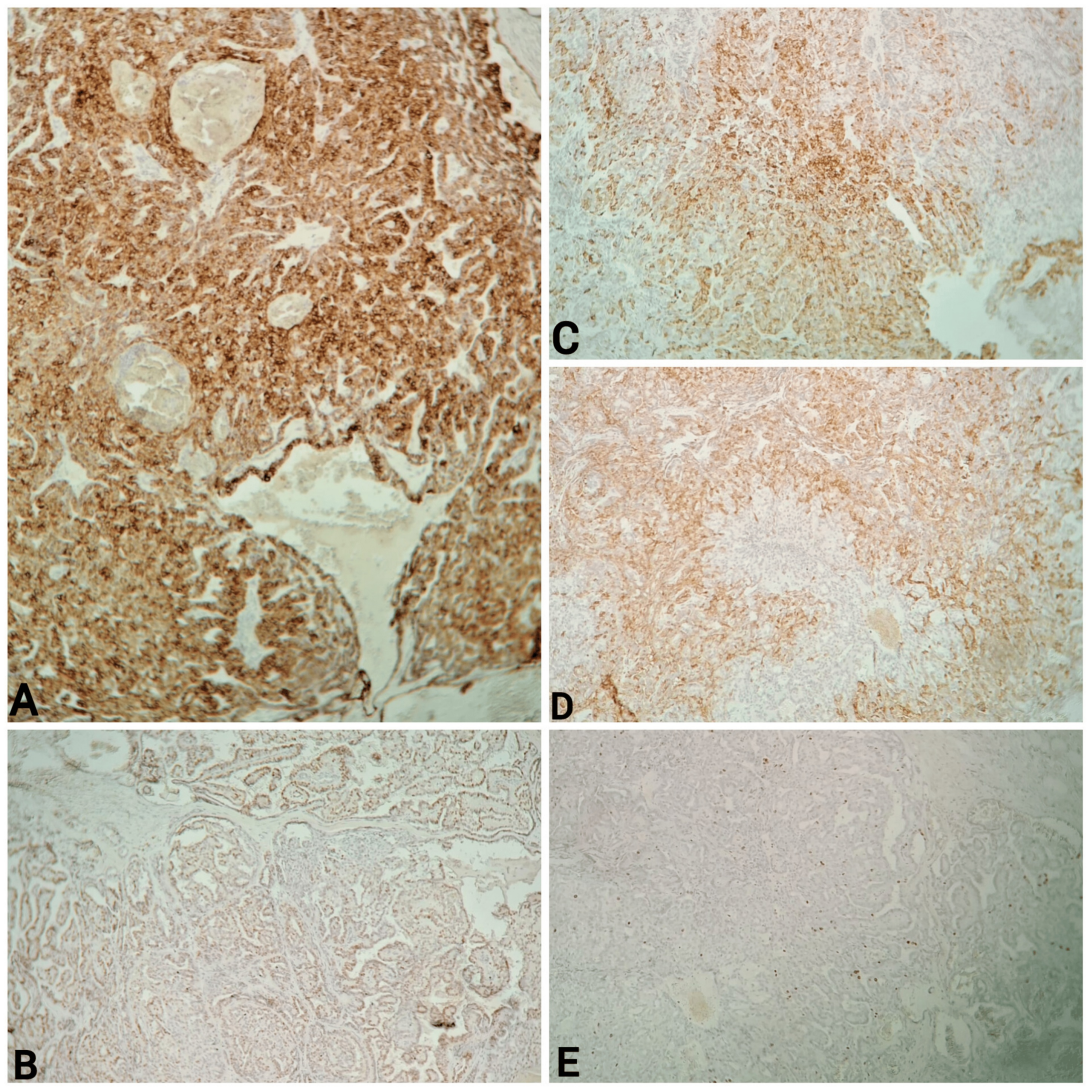
Immunostaining of papillary cystadenoma A: Tumor cells strongly positive for CK7; B: Tumor cell nuclei with a positive reaction for P63; C: Tumor cells positive for AML; D: Tumor cells positive for GCDFP-15; E: Ki-67 proliferation index was low (5%)

The surgical margins were measured at 1 mm. Immunohistochemically, the tumor cells were positive for CK7, P63, AML, and GCDFP-15; Ki-67 was low at 5% (Figure 2).

**Figure 3:**
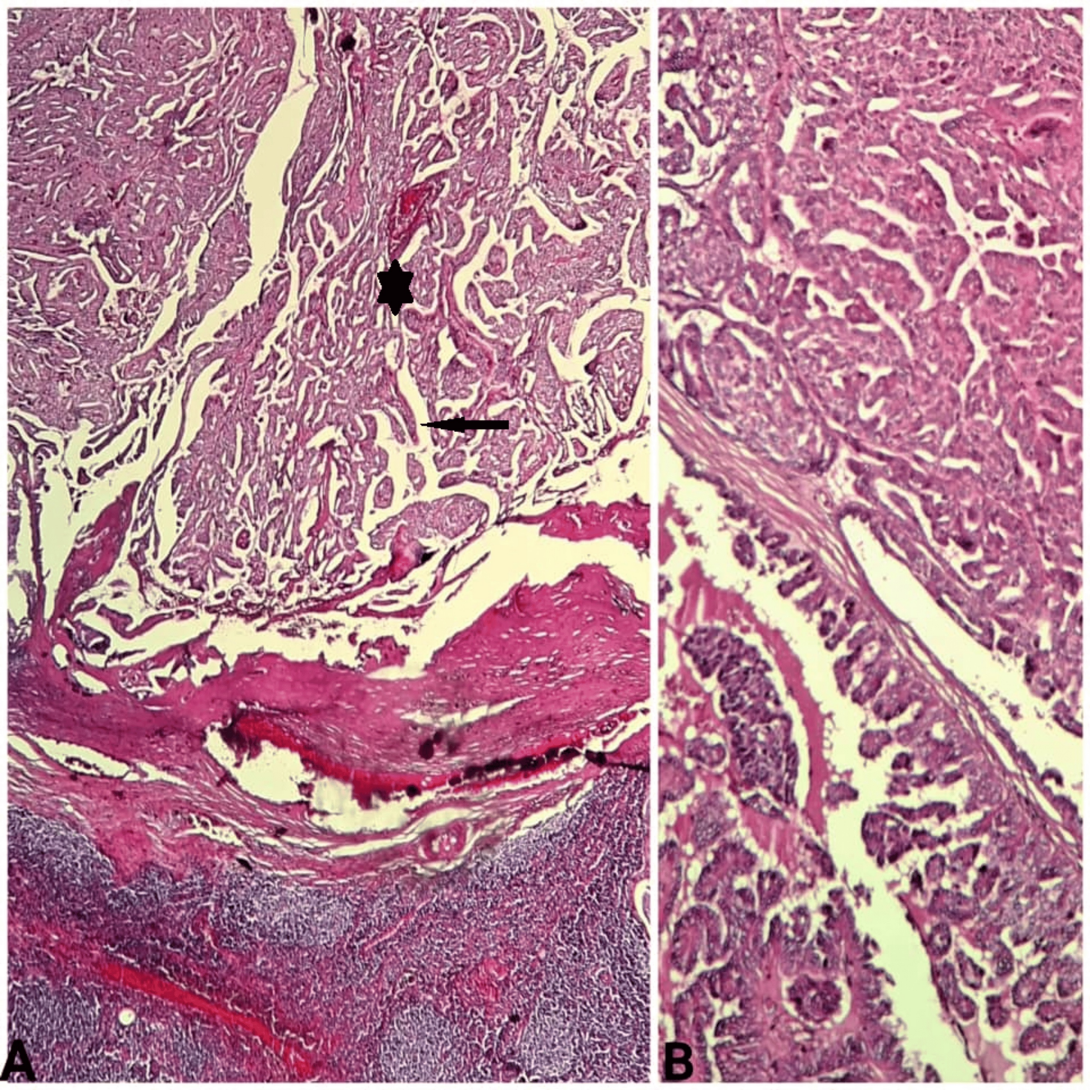
Cervical LN metastasis of papillary cystadenocarcinoma A: Cystic lesion lined with complex papillary fronds (black star) with fibrovascular core (black arrow) (H&E stain, magnification x10); B: A double-cell layer of neoplastic epithelial cells exhibiting large, round, vesicular, and uniform nuclei with inconspicuous nucleoli (H&E stain, magnification x40) LN: Lymph node

Based on the clinical, histological, and immunohistochemical findings, a diagnosis of papillary cystadenoma of the minor salivary gland was established. Two years later, the patient returned with a complaint of painless swelling in the right cervical region, which had persisted for approximately four months. Ultrasonography revealed a multilobulated LN with necrosis and no other masses in the thyroid or parotid glands. An excisional biopsy was performed. Histopathological examination revealed malignant papillary proliferation within the LN (Figure 3).

**Figure 4:**
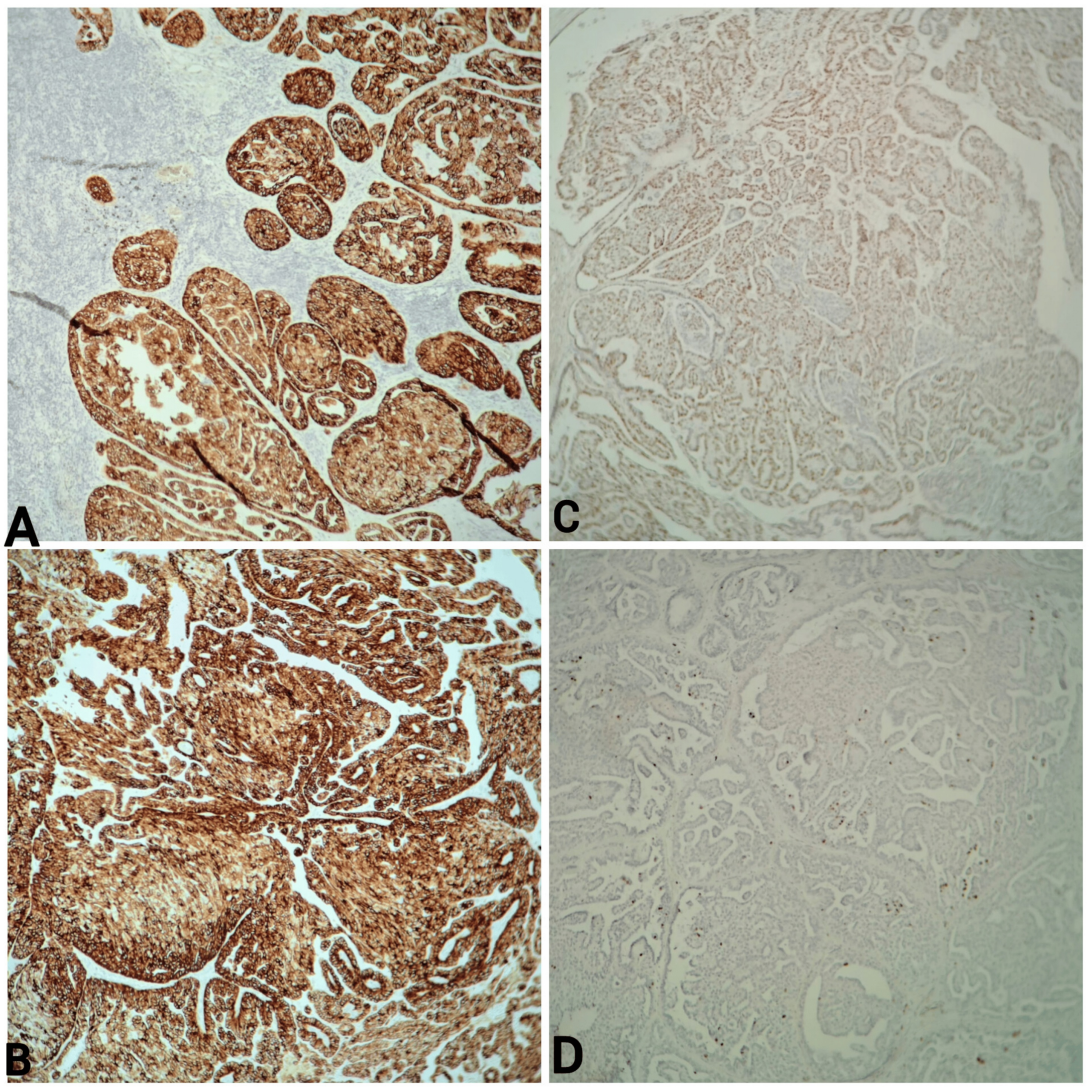
Immunostaining of LN papillary cystadenocarcinoma A: Tumor cells strongly positive for CK7; B: Tumor cells positive for GCDFP-15; C: Tumor cell nuclei with a positive reaction for P63; D: Ki-67 proliferation index was low at 5%

Immunohistochemically, the tumor cells were positive for CK7, P63, AML, and GCDFP-15, and Ki-67 was estimated at 5% (Figure 4), and negative for the androgen receptor (AR), CK20, thyroglobulin, TTF1, estrogen receptor (ER), and progesterone receptor (PR).

Based on the medical history, absence of another primary cancer on CT imaging, and histological and immunohistochemical analyses, we concluded that the cervical LN was involved by a low-grade papillary cystadenocarcinoma of the salivary gland. Examination of the oral cavity revealed a small nodule measuring approximately 0.8 mm × 0.5 mm on an old scar. At a multidisciplinary team meeting, it was decided that an excisional biopsy of the nodule, followed by neck dissection, should be performed.

Histological examination of the nodule confirmed low-grade papillary cystadenocarcinoma of the salivary gland. The surgical margins were also clear. Histological examination of the neck dissection specimen revealed seven LNs, three of which were metastatic. The combined clinical history and morphologic (papillary-cystic component) with histologic evidence of LN invasion and immunohistochemical profile (strong positivity for CK7, P63, AML, and GCDFP-15) support the final diagnosis of cystadenocarcinoma and allowed exclusion of major differential diagnoses such as papillary cystadenoma, mucoepidermoid carcinoma, and salivary duct carcinoma. Given the rarity and limited evidence for this specific subtype, decisions regarding systemic therapy were made in a multidisciplinary setting, considering adjuvant radiotherapy.

## Discussion

Papillary cystadenocarcinoma of the salivary glands is extremely rare and typically low-grade [4]. Differentiating papillary cystadenoma from cystadenoma can be challenging in certain cases. The fifth edition (2022) of the World Health Organization (WHO) Classification of Tumors of the Head and Neck cautiously addresses this issue by categorizing these tumors as non-specific adenocarcinomas [5]. In particular, histological differences, such as capsule infiltration and/or pronounced cytonuclear atypia [5], as well as vascular and perineural invasion [6], can be difficult to determine. Furthermore, no immunohistochemical markers currently exist to definitively determine malignancy. In the present case, although the entire nodule was submitted and examined, no capsular or vascular invasion was observed in the specimen. Malignant cytonuclear features were absent, even in the metastatic LNs. A continuum of lesions between these two entities has also been proposed. However, there is no conclusive evidence, and molecular data are insufficient to confirm the benign-malignant sequence [7,8].

Owing to the papillary nature of the tumor, it was crucial to confirm that the metastasis originated from the primary salivary gland. This was achieved through clinical, radiological, and immunohistochemical examinations to rule out papillary adenocarcinomas of the thyroid, kidney, breast, and ovaries [9]. The absence of AR leads to the exclusion of ductal adenocarcinoma of the salivary glands [10]. Other papillary adenocarcinomas, such as those from the thyroid and lung, were ruled out based on TTF1 negativity, whereas those from the breast and ovaries were excluded because of ER and PR negativity [11].

Recurrent tumors should be considered in cases of papillary cystadenocarcinoma of the salivary glands, even in those classified as low-grade. These cells have a distinct metastatic potential. Although instances of recurrence and metastasis, particularly to the cervical LN, have been documented, they remain relatively uncommon [12]. The initial 1 mm surgical margin for the presumed cystadenoma, although considered clear, may be interpreted as narrow. Given the subsequent development of metastatic cystadenocarcinoma, this initial margin likely contributes to the persistence or recurrence of the tumor, thereby highlighting the importance of achieving wider margins in such cases, even for lesions initially deemed benign. This finding reinforces the literature, indicating that narrow margins are a significant risk factor for recurrence of salivary gland tumors, even those initially presenting as low-grade or benign-appearing [13]. Recurrence appears to be more strongly associated with narrow surgical margins than multifocality [14,15]. A study involving 40 patients reported a metastasis rate of 22.5% [2]. The tendency for cervical LN metastases may be attributed to the rich lymphatic network present in the oral mucosa [6].

As with most salivary gland carcinomas, there is currently no consensus on the management of either the localized or metastatic forms [16]. This lack of agreement can be attributed to the rarity of these tumors, which constitute only 5% of all salivary gland cancers ​[17]. Consequently, owing to the risk of recurrence and metastatic spread, some studies advocate for extensive surgical excision combined with systematic ipsilateral cervical LN dissection, even when no LN metastasis is present in the staging assessment [15]. Additionally, adjuvant radiotherapy or systemic therapy is recommended for patients with poor prognostic factors, such as adverse pathological features (intermediate- or high-grade, close or positive margins, neural or perineural invasion, LN metastases, and lymphatic or vascular invasion) [13]. However, its effects on survival remain unclear [18].

## Conclusions

Despite advancements in immunohistochemistry and molecular biology, distinguishing between malignant and benign cystadenomatous tumors of salivary glands remains a challenge. This complexity is exemplified in this case report of a 58-year-old woman, as evidenced by her medical history and pathological findings. These tumors have the potential to recur and metastasize, even in low-grade forms. Therefore, all the specimens should be thoroughly examined. Patients diagnosed with salivary gland cystadenomas should be closely monitored for early signs of relapse or metastasis. Therefore, understanding these pathological nuances is essential for clinicians and pathologists. Analyzing the molecular differences between the localized and metastatic forms of this neoplasm would enhance understanding and documentation of this exceptional case.
